# Embryonic scaling: morphogen gradients, size sensing, and scaler genes

**DOI:** 10.3389/fcell.2026.1858676

**Published:** 2026-06-17

**Authors:** Polina S. Timoshina, Andrey G. Zaraisky

**Affiliations:** 1 Shemyakin-Ovchinnikov Institute of Bioorganic Chemistry, Russian Academy of Sciences, Moscow, Russia; 2 Koltzov Institute of Developmental Biology, Russian Academy of Sciences, Moscow, Russia

**Keywords:** embryonic patterning, morphogen gradient, reaction diffusion, scaler genes, scaling, sea urchin, *Xenopus*

## Abstract

Embryonic scaling is the ability of developing embryos to preserve proportional patterning despite differences in overall size. This phenomenon has long been recognized in many animal groups and represents a central example of developmental robustness and self-regulation. Its mechanisms are now being clarified through quantitative embryology, theoretical modeling, molecular developmental biology, and mechanobiology. The best-characterized framework for understanding embryonic scaling is morphogen-gradient scaling. In this view, pattern proportions are preserved when morphogen gradients adjust their range and threshold positions according to the size of the embryo or morphogenetic field. Several mechanisms can contribute to this adjustment, including feedback regulation within morphogen networks, size-dependent morphogen production or degradation, ligand transport, and mechanical or geometric constraints. Earlier theoretical studies anticipated the possibility that morphogen-gradient scaling may require size-dependent modulators, that is, regulatory components whose concentration or activity changes with system size. This review considers such size-dependent regulation in the context of the Scalers Hypothesis, which focuses on experimentally identifiable genes and proteins whose expression, concentration, or activity changes systematically with embryo size. Their products can therefore act as molecular links between global geometry and local patterning dynamics. In this way, they may adjust morphogen production, degradation, transport, diffusion range, or threshold interpretation in a size-dependent manner. The review places scaler genes within a broader comparative framework that includes *Drosophila* BMP/Dpp-Sog and Bicoid systems, chick blastoderm regulation, mechanical models of epithelial patterning, and regenerative or synthetic systems such as Hydra, planarians, and gastruloids. It then distinguishes between internal scalers, which are embedded within morphogenetic feedback networks such as BMP-Chordin and Nodal-Lefty systems, and external scalers, whose regulation is independent of the core pattern-forming circuitry. Finally, the review summarizes experimental evidence from amphibian and echinoderm embryos showing that matrix metalloproteinase Mmp3 in *Xenopus laevis* and the astacin-like proteases Span and Bp10 in sea urchins function as external scalers required for size-dependent adjustment of BMP-Chordin gradients. These findings support a modular view of embryonic scaling, in which size sensing and pattern generation may be partially separable. They also suggest practical strategies for identifying size-dependent regulators in other embryos, organ primordia, regenerative systems, and engineered multicellular models.

## Introduction

**FIGURE 1:**
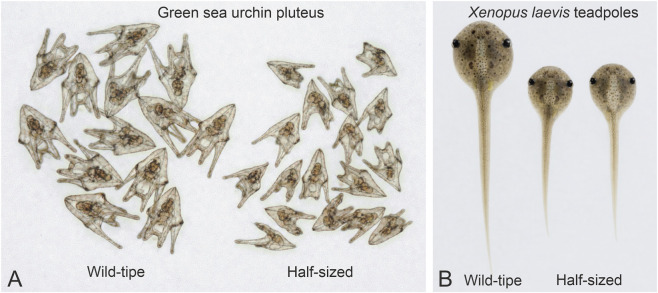
Embryonic scaling: proportional development despite size variation. Wild-type embryos and proportionally smaller “half-sized” twins obtained by separation of the first two blastomeres according to the methods described in Orlov et al. (2025) **(A)** Pluteus larvae of the green sea urchin *Strongylocentrotus droebachiensis*. **(B)** Tadpoles of the frog *Xenopus laevis*. In both species, embryos derived from individual blastomeres develop into morphologically normal but reduced-size organisms. These examples illustrate scale invariance in embryonic development, i.e., preservation of body proportions and axial patterning despite substantial changes in overall embryo size. Scale bars: **(A)** 150 μm; **(B)** 500 μm.

During embryonic development, spatial patterning establishes ordered cell domains that later give rise to tissues and organs. A central question in developmental biology is how this spatial organization remains proportionally correct when the overall size of the embryo changes (see two examples of embryonic scaling in Figure 1). In many animals, embryos can develop into well-proportioned organisms even when their absolute dimensions differ substantially. This phenomenon is known as embryonic scaling, or embryonic self-regulation. It reflects the ability of the early embryo to behave as an integrated morphogenetic field, in which individual regions develop according to their position within the whole embryo rather than according to absolute distance alone (De Robertis, 2006; Ben-Zvi et al., 2008).

Here, scale invariance means that relative pattern proportions are preserved when the absolute size of the embryo or patterning field changes. For example, if a particular cell-fate domain occupies a given fraction of an embryonic axis in a normal embryo, it should occupy approximately the same fraction of that axis in a smaller or larger embryo. Thus, scaling does not mean that all structures keep the same absolute dimensions. Rather, it means that the spatial pattern is resized in proportion to the whole system.

Experimental evidence for embryonic scaling dates back to the late 19th century, when Hans Driesch showed that isolated blastomeres from two-cell and four-cell sea urchin embryos can develop into smaller but proportionally normal larvae (Driesch, 1891). At the same time, regulative scaling is not unlimited. Classic sea urchin blastomere experiments showed that animal and vegetal blastomere tiers separated at the 8-cell stage do not behave simply as equivalent scaled-down embryos. We now use this example to emphasize that successful scaling depends not only on reduction in size, but also on preservation or reconstruction of the relevant axial information and patterning capacity (Hörstadius, 1939; Cameron et al., 1987).

Since then, scaling and regulative development have been documented in many animal groups.Examples include cnidarians such as *Nematostella vectensis* (Fritzenwanker et al., 2007), insects such as *Drosophila melanogaster* (Cheung et al., 2011; Garcia et al., 2013; He et al., 2015), fish (Almuedo-Castillo et al., 2018), amphibians (Morgan, 1895; Spemann and Mangold, 1924; Ben-Zvi et al., 2008; Inomata et al., 2013; Orlov et al., 2022), birds (Spratt and Haas, 1960; Spratt and Haas, 1961; Khaner, 1996; Bertocchini and Stern, 2002), and mammals (Katayama et al., 2010; Hashiyada, 2017). Scaling is also central to regeneration, where organisms such as *Hydra* and planarians can restore complete and proportionally patterned body axes from fragments of different sizes (Bode and Bode, 1980; Hill and Petersen, 2015).

Importantly, scaling is not only a response to extreme experimental manipulations. Embryos also vary in size under natural conditions. Such variation may arise from differences in egg size, yolk content, maternal provisioning, maternal physiological state, clutch-to-clutch variation, or species-specific reproductive strategies. In amphibians, including *Xenopus*, differences in embryo size are likely to be established largely during oogenesis, through variation in oocyte growth, yolk accumulation, and maternal provisioning, although the precise causes of embryo-to-embryo size variation within a single clutch remain incompletely understood. These sources of variation create a biological need for patterning systems that remain robust despite differences in initial embryo geometry. In *Xenopus laevis*, embryos from a single clutch may vary in diameter by up to 38%, corresponding to more than a twofold difference in volume, yet still develop into proportionally normal tadpoles with appropriately scaled gene-expression domains (Leibovich et al., 2020). Thus, embryonic scaling is relevant not only to experimental embryology, but also to natural developmental variation and evolutionary changes in egg and embryo size.

For much of the 20th century, the phenomenon of embryonic scaling was well known, but its mechanisms remained obscure. A major conceptual advance came from theories of self-organization and reaction–diffusion pattern formation. Turing proposed that spatial patterns can arise spontaneously through interactions between locally acting and more broadly spreading components (Turing, 1952). Gierer and Meinhardt later developed related models in which stable patterns emerge from the balance between production, diffusion, interaction, and degradation of molecular components (Gierer and Meinhardt, 1972). In parallel, Wolpert’s concept of positional information explained how cells can read their position by responding to different threshold concentrations of a graded signal (Wolpert, 1969). Together, these ideas established morphogen gradients as a central framework for understanding embryonic patterning.

Within this framework, embryonic scaling can be understood as a problem of proportional morphogen-gradient rescaling. If a morphogen gradient adjusts to embryo size, then the positions of threshold concentrations shift proportionally with the size of the patterning field. As a result, cell-fate boundaries remain at the correct relative positions. If the gradient does not scale correctly, the same thresholds will be reached at inappropriate positions, and the resulting pattern will be distorted. Thus, scaling requires not only the formation of a morphogen gradient, but also the adjustment of that gradient to the size and geometry of the embryo.

Several types of mechanisms have been proposed to explain how such adjustment may occur. These include boundary-condition models, annihilation-based models, external modulator models, and feedback-based models (Umulis and Othmer, 2013; Čapek and Müller, 2019). Feedback-based models have received especially strong experimental support. In these systems, regulatory interactions within the patterning network itself help adjust the range or activity of a morphogen signal. Important examples include the *Drosophila* BMP/Dpp–Sog/Tsg–Tld system, which established key principles of BMP ligand shuttling, protease-dependent ligand release, and gradient robustness (Marqués et al., 1997; Eldar et al., 2002; Shimmi et al., 2005; Mizutani et al., 2005; Umulis et al., 2006; 2010); the BMP–Chordin–ADMP/Sizzled system in *Xenopus laevis*, in which feedback regulation adjusts Chordin range and BMP activity to embryo size (Ben-Zvi et al., 2008; Ben-Zvi and Barkai, 2010; Inomata et al., 2013); and the Nodal–Lefty system in zebrafish, in which size-dependent inhibition restricts the Nodal signaling range (Almuedo-Castillo et al., 2018).

At the same time, embryonic scaling should not be reduced exclusively to biochemical morphogen gradients. Mechanical and geometric factors may also contribute to size-dependent patterning. These include tissue curvature, tension, cell packing, hydrostatic pressure, extracellular matrix organization, and boundary constraints. Recent work in avian embryos has shown that tissue mechanics can self-organize through feedback between local contractility and long-range tension, thereby influencing tissue flows and gene expression during embryonic regulation (Caldarelli et al., 2024). This modern view has historical precedents in physical models of epithelial morphogenesis based on elastic forces and contact cell polarization, which showed that epithelial sheets can subdivide into proportionally scaled domains through mechanical interactions alone (Belintsev et al., 1985a; b, 1987). Thus, biochemical, mechanical, and geometric processes may cooperate in producing scale-invariant patterns.

Despite this diversity of possible mechanisms, studies of morphogen-gradient scaling point to a common principle. Correct scaling usually requires at least one regulatory variable that changes with the size of the patterning field. Related theoretical concepts had been discussed previously under the term “modulators” and in external modulator models, in which size-dependent regulatory factors influence reaction-diffusion dynamics (Umulis and Othmer, 2013; Rasolonjanahary and Vasiev, 2016). A similar logic is also present in feedback-based models, where components such as expanders or inhibitors change the effective range of a morphogen gradient (Ben-Zvi and Barkai, 2010; Almuedo-Castillo et al., 2018). These studies suggest that size-dependent regulatory components are not secondary details, but may be essential parts of systems capable of accurate proportional scaling.

This reasoning led to the formulation of the Scalers Hypothesis in a series of theoretical and experimental studies (Nesterenko and Zaraisky, 2019; Orlov et al., 2022; Timoshina et al., 2024). According to this hypothesis, accurate morphogen-gradient scaling requires regulators whose expression, concentration, or activity changes systematically with embryo size. The genes encoding such regulators were defined as scaler genes, or scalers. Their distinctive feature is that their expression is sensitive to the geometric size of the embryo or morphogenetic field. As a result, the concentrations or activities of their products change with system size, unlike scale-invariant components whose local concentrations remain approximately unchanged in correctly scaled embryos. In this way, scaler genes serve as molecular links between global geometry and local patterning dynamics. By changing their expression or activity in a size-dependent manner, they regulate morphogen production, degradation, transport, diffusion range, or threshold interpretation according to embryo size.

Formal mathematical analysis supports this view. In closed reaction–diffusion systems with zero-flux boundary conditions, proportional scaling cannot be achieved if all internal dynamic components remain scale-invariant (Timoshina et al., 2024; see Supplementary Text S2 for the proof of the scaling lemma). In such systems, at least one component must vary with system size. In real embryos, which are not strictly closed systems, size-dependent regulation may be provided either by components embedded within the pattern-forming network or by external regulators that convey size information to it. This distinction provides the basis for separating internal scalers, which are part of the morphogenetic feedback network, from external scalers, which regulate that network from outside.

The present review synthesizes theoretical and experimental advances that have clarified the role of size-dependent regulators in embryonic patterning. Its aim is not to provide an exhaustive catalogue of all known scaling phenomena, but to focus on a specific mechanistic question: how information about system size is coupled to morphogen-gradient formation and proportional patterning. To place this question in a broader context, the review first discusses major theoretical models and experimental examples of scaling, including *Drosophila* BMP/Dpp–Sog and Bicoid systems, chick blastoderm regulation, mechanical models of epithelial patterning, and regenerative or synthetic systems such as *Hydra*, planarians, and gastruloids. It then focuses on molecular size-dependent regulators, or scalers, and discusses the distinction between internal scalers and external scalers. This framework allows experimentally identified scaler genes in *Xenopus* and sea urchin embryos to be considered as case studies within the broader problem of size-dependent patterning.

## Mechanisms of embryonic scaling

### Morphogen gradients and the scaling problem

Embryonic scaling refers to the ability of an embryo or morphogenetic field to preserve relative pattern proportions despite changes in absolute size. In a scaled system, a cell fate domain that occupies a given fraction of an axis in a normal embryo occupies approximately the same fraction of that axis in a smaller or larger embryo. This property can be described as scale invariance: the absolute dimensions of the pattern change, but the relative positions of its main boundaries are preserved.

Several types of mechanisms may contribute to such size-dependent patterning. The best-characterized framework is based on morphogen gradients, in which cells interpret local concentrations of signaling molecules relative to defined thresholds (Wolpert, 1969; Ashe and Briscoe, 2006; Lander, 2007; Rogers and Schier, 2011). If the spatial range of a morphogen gradient changes in proportion to the size of the patterning field, threshold-dependent domains of gene expression and differentiation can retain their relative positions. For this reason, embryonic scaling has often been formulated as a problem of morphogen-gradient scaling.

At the same time, proportional patterning need not depend exclusively on biochemical gradients. Embryos and tissues also differ in curvature, surface-to-volume ratio, tissue tension, hydrostatic pressure, cell packing, extracellular matrix organization, and boundary constraints. These physical parameters may influence patterning indirectly, by modulating signaling pathways and gene expression, or directly, by altering tissue flows, cell rearrangements, extracellular diffusion, morphogen transport, receptor accessibility, or the geometry of the patterning field (Kicheva et al., 2012; Hannezo and Heisenberg, 2019; Goodwin et al., 2021; Caldarelli et al., 2024). Thus, mechanical and geometric mechanisms may cooperate with morphogen gradients, and in some contexts may themselves provide size-dependent information. Nevertheless, because morphogen-gradient scaling is currently the most extensively developed theoretical and experimental framework for analysing embryonic scaling, it provides the starting point for the discussion below.

The conceptual foundations of morphogen-based patterning trace back to the work of Turing, Wolpert, and Gierer and Meinhardt. Turing proposed that spatial patterns can emerge spontaneously from an initially homogeneous state through local self-activation coupled with longer-range inhibition (Turing, 1952). Wolpert’s positional information model then established how graded distributions can be interpreted by cells through concentration thresholds to generate discrete cell fates (Wolpert, 1969). Gierer and Meinhardt formalized related principles within a reaction-diffusion framework, showing how stable spatial patterns can arise from the dynamic balance between production, diffusion, interaction, and degradation of molecular components (Gierer and Meinhardt, 1972).

**FIGURE 2:**
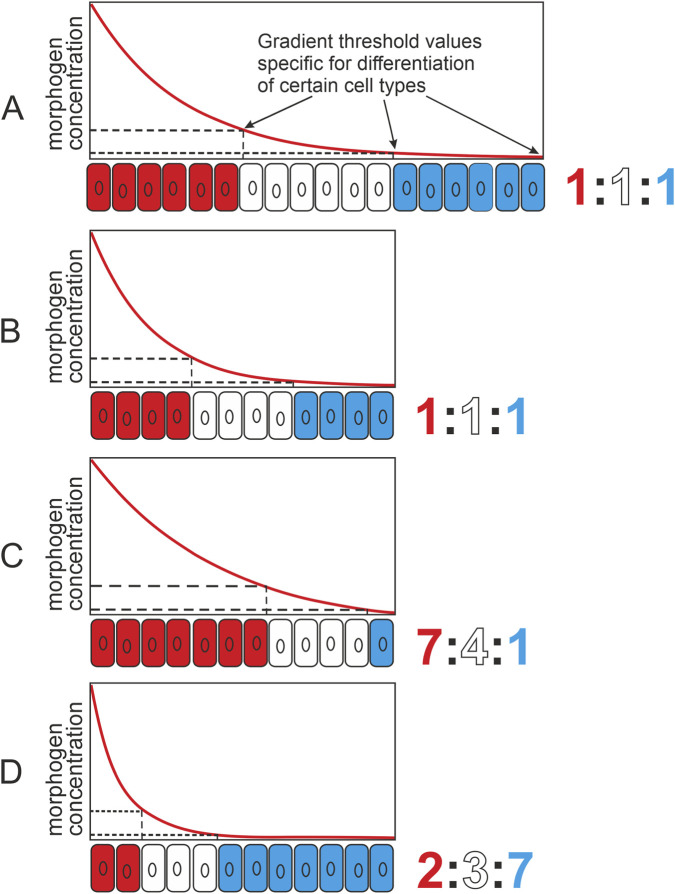
Correct and incorrect scaling of morphogen gradients **(A)** Morphogen gradient in an original system of length L generating three threshold-defined cell domains. **(B)** Proportionally rescaled gradient in a reduced system, preserving relative threshold positions and pattern proportions. **(C,D)** Improperly scaled gradients in a reduced system leading to hyper-scaling or hypo-scaling. Only proportional threshold rescaling maintains invariant spatial patterning.

Within this framework, embryonic scaling can be formulated as a problem of proportional gradient rescaling. If morphogen distributions adjust appropriately to changes in system size, the relative positions of threshold concentrations remain invariant, and spatial pattern proportions are preserved (Figures 2A,B). By contrast, if the gradient contracts too strongly or not enough, threshold positions shift disproportionately, leading to hyper-scaling or hypo-scaling and to distorted differentiation domains (Figures 2C,D).

Although Figure 2 emphasizes changes in gradient shape and range, proportional patterning could also be supported by size-dependent changes in morphogen source strength, gradient amplitude, degradation rate, transport, receptor accessibility, or cellular response thresholds. Thus, Figure 2 should be viewed as a schematic illustration of one central aspect of scaling, rather than as an exhaustive representation of all possible scaling mechanisms.

Experimental examples corresponding to the non-proportional scaling scenarios shown schematically in Figure 2C and D have been described in several morphogen systems.

Perturbation or size-dependent modulation of morphogen-gradient formation can shift threshold positions and alter embryonic patterning, as shown for Chordin/BMP-mediated dorsal-ventral patterning in *Xenopus* and sea urchin embryos (Inomata et al., 2013; Orlov et al., 2022; Timoshina et al., 2024), BMP/Dpp-Sog-mediated dorsal patterning in *Drosophila* (Marques et al., 1997; Eldar et al., 2002; Shimmi et al., 2005; Mizutani et al., 2005), Bicoid-dependent anterior-posterior patterning in *Drosophila* (Driever and Nüsslein-Volhard, 1988a; b), and Nodal-Lefty-dependent mesendoderm patterning in zebrafish (Almuedo-Castillo et al., 2018).

### Theoretical models of morphogen-gradient scaling

Not all reaction-diffusion systems automatically produce such proportional rescaling. Classical Turing-type and Gierer-Meinhardt-type systems can generate stable spatial patterns, but they do not necessarily preserve relative pattern proportions when system size changes. This limitation led to the development of theoretical models specifically addressing how morphogen gradients can adjust to global geometry. These models can be grouped into four broad classes: boundary-condition scaling, annihilation-based scaling, external modulator scaling, and feedback-based scaling mechanisms (Umulis and Othmer, 2013; Čapek anf Müller, 2019).

The first class, boundary-condition scaling, assumes that morphogen concentrations are fixed at opposite ends of the morphogenetic field, with a maximum at the source and a minimum at the distal boundary. Under such idealized conditions, a gradient can change its spatial profile in proportion to field size. However, strict boundary conditions are difficult to realize biologically. A localized source of morphogen production is common, but a fixed distal concentration is less straightforward to maintain under continuous diffusion, degradation, and tissue growth. As a result, deviations from proportional scaling may arise, especially near the distal end of the field (Umulis and Othmer, 2013).

The second class, annihilation-based scaling, invokes two opposing gradients whose components mutually reduce or inactivate one another (McHale et al., 2006; Rasolonjanahary and Vasiev, 2016). Such interactions can, in principle, place pattern boundaries at positions determined by the balance between opposing signals. Opposing anterior-posterior maternal systems in *Drosophila*, including Bicoid and posterior determinants such as Nanos, have often been discussed in relation to this type of model, although their biological interactions are more indirect than simple chemical annihilation. Mathematical analyses suggest that annihilation-like mechanisms can contribute to scaling, but accurate proportional scaling often still requires boundary constraints or additional regulatory inputs (Rasolonjanahary and Vasiev, 2016).

The third class, external modulator scaling, proposes that a morphogen gradient can be scaled by an additional factor produced independently of the core gradient-generating reaction-diffusion system. Such a factor may acquire a size-dependent concentration or spatial profile and then influence morphogen degradation, transport, or availability. This idea is important because it separates size sensing from the pattern-forming gradient itself. The possibility that size-sensitive modulators can regulate morphogen scaling was developed theoretically by Umulis and Othmer (2013) and by Rasolonjanahary and Vasiev (2016). Thus, the general concept of an external size-dependent regulator has important precedents in theoretical work; the question addressed by later experimental studies is whether such regulators can be identified as concrete molecular components in real embryos.

The fourth class, feedback-based scaling, has received particularly strong experimental support and is rooted in a broader literature on feedback-controlled morphogen-gradient formation and robustness. In these models, scaling or robustness emerges from regulatory interactions embedded within the morphogenetic network itself. Such feedback architectures allow local molecular interactions - such as ligand transport, inhibitor-mediated shuttling, proteolytic release, and transcriptional feedback - to generate a system-level response to changes in field size or molecular dosage. Important examples include the *Drosophila* BMP/Dpp-Sog/Tsg-Tld system, which established key principles of BMP ligand shuttling, protease-dependent ligand release, and gradient robustness; the BMP-Chordin-ADMP/Sizzled system in *Xenopus laevis*, in which feedback regulation adjusts Chordin range and BMP activity to embryo size; and the Nodal-Lefty system in zebrafish, in which size-dependent inhibition contracts the Nodal signaling range (Eldar et al., 2002; Shimmi et al., 2005; Mizutani et al., 2005; Umulis et al., 2006; 2010; Ben-Zvi et al., 2008; Ben-Zvi and Barkai, 2010; Inomata et al., 2013; Almuedo-Castillo et al., 2018).

### Experimental examples of morphogen-based scaling

One of the earliest and most influential examples of quantitative modeling of an extracellular BMP-type patterning system comes from dorsal-ventral patterning in *Drosophila*. In this system, dorsal patterning depends on the BMP ligands Decapentaplegic (Dpp) and Screw (Scw), the BMP antagonist Short gastrulation (Sog), the Sog-associated protein Twisted gastrulation (Tsg), and the metalloprotease Tolloid (Tld). This network is closely analogous to vertebrate BMP-Chordin systems: Sog is the functional counterpart of Chordin, and Tld cleaves Sog-containing complexes to release BMP ligands. Early experimental work showed that Dpp activity in the dorsal ectoderm is shaped by the opposing activities of Sog and Tld, and that BMP signaling becomes refined into a dorsal peak marked by phosphorylated Mad (Marques et al., 1997; Dorfman and Shilo, 2001).

A major theoretical and experimental advance was made by Eldar and colleagues, including Barkai, who showed that the BMP activation gradient in *Drosophila* embryonic patterning is robust to changes in gene dosage and that Sog-mediated facilitated transport can contribute to this robustness (Eldar et al., 2002). Subsequent studies developed this mechanism further, showing that Sog/Tsg-mediated transport of Dpp/Scw complexes and Tld-dependent cleavage are central for sharpening and stabilizing the dorsal BMP signaling peak (Shimmi et al., 2005; Mizutani et al., 2005; Umulis et al., 2006). Later organism-scale modeling incorporated embryo geometry and quantitative BMP signaling data to analyse early *Drosophila* BMP-mediated patterning at the scale of the whole embryo (Umulis et al., 2010). This literature is important in the present context because it predates many later vertebrate BMP-Chordin scaling models and established several principles that recur in them: facilitated ligand transport by an antagonist, protease-dependent ligand release, robustness to parameter variation, and adjustment of BMP signaling domains to embryo geometry.

A related feedback-based mechanism was later analysed in the dorsal-ventral axis of size-reduced *Xenopus laevis* embryos derived from dorsal half-gastrulae (Ben-Zvi et al., 2008). Dorsal-ventral patterning in *Xenopus* is established through antagonism between ventrally active BMP signaling and its dorsal inhibitor Chordin, secreted by the Spemann organizer. The organizer also expresses ADMP, a BMP-like ligand whose transcription is negatively regulated by BMP signaling. In dorsal half-embryos that retain the organizer but lack the original ventral region, the system must regenerate a ventral domain with high BMP activity and low Chordin activity.

Modeling and experimental analyses showed that this reconstruction depends on an integrated feedback circuit involving BMP, Chordin, and ADMP. Chordin binds BMP and inhibits BMP signaling, while also promoting long-range ligand movement through shuttling. ADMP provides an additional BMP source when BMP signaling is low. As BMP activity increases, it represses both Chordin and ADMP expression. This feedback architecture, termed the expansion-repression model, enables proportional adjustment of the BMP activity gradient to changes in embryo size (Ben-Zvi and Barkai, 2010).

Further experimental work identified Sizzled as an additional component of this scaling mechanism (Inomata et al., 2013). Sizzled is induced by BMP signaling and inhibits Tolloid-family metalloproteases that cleave Chordin. By limiting Chordin degradation, Sizzled stabilizes Chordin and increases its effective range. In reduced embryos, Sizzled levels decrease, Tolloid activity increases, the Chordin gradient contracts, and the BMP activity gradient is rescaled accordingly. This mechanism illustrates how feedback within a morphogenetic network can generate size-dependent adjustment without requiring an independent geometric ruler.

A distinct feedback-based mechanism operates in zebrafish embryos during Nodal-mediated mesendoderm induction. Nodal promotes mesendoderm formation and induces expression of Lefty, a rapidly diffusing inhibitor that restricts Nodal signaling over long distances. Almuedo-Castillo et al. described this system as an induction-contraction mechanism (Almuedo-Castillo et al., 2018). When part of the blastoderm is removed, the reduced embryonic volume leads to an increased effective concentration of Lefty, which contracts the Nodal signaling range and restores proportional patterning. Thus, whereas the *Xenopus* BMP-Chordin system achieves scaling through regulated expansion and repression, the zebrafish Nodal-Lefty system achieves scaling through size-dependent restriction of signaling range.

Other systems show that scaling can also arise through mechanisms upstream or downstream of a morphogen gradient. A particularly well-studied example is the anterior-posterior Bicoid system in *Drosophila*. Bicoid forms a nuclear protein gradient from the anterior pole and provides positional information for anterior patterning. Quantitative studies have shown that Bicoid gradient properties can scale with embryo dimensions. Cheung et al. proposed that within-species scaling of the Bicoid gradient can be achieved through a volume-dependent production rate, linking maternal bicoid mRNA input and embryo volume to the amplitude of the protein gradient (Cheung et al., 2011). Subsequent work further suggested that Bicoid scaling is constrained by coordinated scaling of maternal tissue and bicoid gene-copy number during oogenesis (He et al., 2015). Thus, in this system, size dependence may enter upstream of the zygotic patterning network, at the level of maternal morphogen production or loading.

In addition to the extracellular BMP/Dpp-Sog system, the earlier maternal Toll-Dorsal system provides a complementary example of size-dependent patterning at the level of a nuclear transcription-factor gradient. Dorsal, an NF-kappaB-related transcription factor, forms a ventral-to-dorsal nuclear gradient and activates target genes at different concentration thresholds. Quantitative analysis of embryos of different sizes showed that the width of the nuclear Dorsal gradient correlates with dorsal-ventral axis length and that target-gene boundaries respond to embryo size in distinct ways (Garcia et al., 2013). This system is important because it shows that scaling may occur not only through proportional rescaling of an upstream extracellular gradient, but also through size-dependent interpretation by downstream enhancers and gene-regulatory networks.

Recent quantitative analysis of early *Drosophila* gene-expression patterns further showed that both discrete positional markers and graded expression profiles scale with embryo length with high precision. This suggests that scale invariance in the early fly embryo is not limited to a single morphogen gradient, but may reflect a broader property of the patterning network (Nikolić et al., 2024).

A classic example of regulative scaling in an amniote embryo is also provided by the chick blastoderm. Spratt and Haas showed that separated or rearranged regions of the early chick blastoderm can generate smaller but complete embryonic axes (Spratt and Haas, 1960; Spratt and Haas, 1961). Unlike spherical or ellipsoidal embryos, the chick blastoderm is a flat epithelial field, and scaling involves re-establishment of axis position within a reduced or rearranged territory. Later work provided a molecular framework for this regulative capacity. Bertocchini and Stern showed that the chick hypoblast helps position the primitive streak by producing Cerberus, an antagonist of Nodal, Wnt, and BMP signaling; removal of the hypoblast can lead to multiple embryonic axes, indicating that inhibitory interactions help restrict where axis formation is permitted (Bertocchini and Stern, 2002). Thus, chick blastoderm regulation illustrates a scaling strategy based on re-positioning signaling centers and inhibitory fields, rather than simply resizing a pre-existing gradient.

### Mechanical mechanisms of size-dependent patterning

The chick and quail blastoderm has also become an important system for studying mechanical self-organization during embryonic regulation. Recent work by Caldarelli et al. showed that tissue mechanics in avian embryos can self-organize through a feedback architecture analogous to reaction-diffusion systems: local contractility acts as a self-activating component, whereas tissue tension acts as a long-range inhibitor (Caldarelli et al., 2024). This mechanical feedback regulates large-scale tissue flows and gene expression and can explain how one or several well-proportioned embryonic axes arise after perturbation. These findings are directly relevant to embryonic scaling because they show that proportional patterning can be controlled by tissue-level mechanics, not only by diffusible biochemical gradients.

This modern mechanical view has important historical precedents in a physical model of epithelial morphogenesis based on elastic forces and contact cell polarization (Belintsev et al., 1985a; b, 1987). In this model, epithelial cells can locally acquire a polarized mechanical state, and polarized regions generate elastic stresses in the surrounding tissue. These stresses, in turn, restrict the further spread of polarization, creating a regulatory interaction analogous to local activation coupled with long-range inhibition. As a result, an initially homogeneous epithelial sheet can spontaneously subdivide into polarized and non-polarized domains. This prediction is consistent with the frequent subdivision of developing epithelia into territories of polarized and non-polarized cells, with polarized domains often giving rise to placodes and other epithelial structures.

A key feature of this model is that the relative proportions of polarized and non-polarized domains can remain approximately invariant when the size of the epithelial sheet changes. Thus, the model provides a mechanical route to scaling in which the relevant patterning variables are not molecular concentration gradients, but cell polarization, elastic stress, and tissue geometry.

At the same time, an important distinction should be made between scaling in morphomechanical models of this type and scaling based on morphogen-gradient regulation. Morphomechanical models such as those cited above reproduce ideal scaling most naturally for binary patterns consisting of two alternative domains. By contrast, morphogen-gradient scaling models can, in principle, support the proportional scaling of more complex patterns composed of multiple domains, each specified by a different threshold concentration along the morphogen gradient. Thus, mechanical self-organization and morphogen-gradient scaling may represent complementary solutions to the general problem of size-dependent patterning, with different strengths depending on the complexity and structure of the pattern to be scaled.

Mechanical regulation does not necessarily act separately from gene regulation. Mechanical forces generated during morphogenesis can influence signaling pathways and transcriptional programs, thereby coupling tissue deformation to cell differentiation. Experimental and theoretical studies of mechanochemical feedback have shown that mechanical and biochemical processes often form integrated regulatory loops during development (Hannezo and Heisenberg, 2019; Goodwin et al., 2021). In *Xenopus laevis*, mechanical perturbations can activate ERK signaling and alter tissue stiffness (Kinoshita et al., 2020), while mechanical tensions in axial tissues have been linked to changes in gene expression (Eroshkin and Zaraisky, 2017; Eroshkin et al., 2024). These findings suggest that mechanical parameters such as tension, curvature, and stiffness may provide size-dependent cues either by directly influencing tissue patterning or by regulating molecular pathways that participate in pattern formation.

### Regenerative and synthetic systems as contexts for scaling

Regeneration provides a distinct context for size-dependent patterning. Unlike experimentally reduced embryos, regenerating systems must reconstruct axes and proportions from fragments that differ in size, shape, polarity, and wound geometry. Scaling in such systems may involve morphogen-like gradients, positional-control genes, organizer re-establishment, tissue remodeling, growth regulation, mechanical constraints, and inherited tissue polarity. Thus, regenerative systems should not be viewed as examples of a single mechanism, but rather as biological contexts in which multiple scaling mechanisms can be integrated.

Hydra and planarians provide classic examples of regenerative size-dependent patterning, but they differ in how explicitly scaling mechanisms have been resolved. In *Hydra*, small tissue fragments can regenerate complete animals with restored axial organization and approximately normal proportions, indicating that axial patterning can be re-established in a field of altered size (Bode, 2003; Vogg et al., 2019). Mechanistically, this process involves reactivation of the head organizer and Wnt/β-catenin signaling. Multiple Wnt genes are expressed during head organizer formation and regeneration, with Wnt3 acting early in this cascade (Lengfeld et al., 2009). Wnt3 expression is localized through a combination of autoregulatory and repressive inputs, providing a mechanism that can restrict organizer formation and prevent inappropriate expansion of head identity (Nakamura et al., 2011). More recent studies further show that generic injury can induce ectopic Wnt organizers and that mechanical oscillations in regenerating tissue spheroids can promote Wnt activation and axial patterning (Cazet et al., 2021; Ferenc et al., 2021). Thus, *Hydra* provides a mechanistically informative example of organizer re-establishment in fragments of different size and geometry. However, the mechanism of scaling in the stricter sense, that is, how the relative proportions of head, body column, and foot are quantitatively adjusted to fragment size, remains less well understood than morphogen-gradient scaling in *Xenopus* and sea urchin embryos.

Planarian regeneration provides a more explicit example of proportional regulation at the organ scale. Planarians can restore body proportions not only by growth after amputation, but also by remodeling existing tissues during degrowth and regrowth. In this system, Wnt signaling promotes posterior identity, whereas anteriorly expressed Notum acts as a Wnt antagonist. Spatial feedback between Wnt and Notum regulates neoblast differentiation and helps adjust brain size relative to body size during reversible growth (Hill and Petersen, 2015). Thus, planarian scaling illustrates how positional information, antagonist-mediated feedback, stem-cell differentiation, and tissue remodeling can be coupled to preserve proportional organ size in animals of different dimensions.

Stem-cell-derived embryoid models, including gastruloids, provide a modern experimental context in which size can be controlled directly. Gastruloids self-organize from aggregates of pluripotent stem cells and can undergo symmetry breaking, axial elongation, and coordinated expression of embryonic patterning genes (van den Brink et al., 2014; Turner et al., 2017; Beccari et al., 2018). Because initial aggregate size and cell number can be experimentally varied, these systems offer a powerful way to test how size affects symmetry breaking, morphogenesis, and gene-expression patterning. Recent quantitative studies have shown that gastruloids can display precise and scalable self-organization, with physical proportions and gene-expression patterns scaling with system size, and that robust morphology and transcriptional profiles are maintained only within a defined size range (Merle et al., 2024; Fiuza et al., 2025). These findings indicate that gastruloids are highly useful for studying size-dependent patterning. However, as in *Hydra*, the underlying mechanisms that sense system size and convert it into proportional patterning remain incompletely resolved. Current evidence suggests that such mechanisms may involve Wnt/Nodal signaling, PCP-dependent elongation, differential cell adhesion or sorting, tissue mechanics, and growth constraints, but their relative contributions remain to be determined.

### From scaling mechanisms to size-dependent molecular regulators

Taken together, these examples show that embryonic and regenerative scaling can be achieved through multiple mechanistic routes. These include proportional adjustment of morphogen gradients, feedback-controlled expansion or contraction of signaling range, size-dependent maternal morphogen production, differential interpretation of transcription-factor gradients, repositioning of signaling centers, self-organized tissue mechanics, growth, and remodeling. Thus, scaling should not be viewed as the product of a single universal mechanism. Rather, it is a general developmental problem that can be solved by different combinations of biochemical, mechanical, geometric, and regulatory processes.

A more specific conclusion emerges from theoretical and experimental studies of morphogen-gradient scaling. In such systems, proportional scaling usually requires at least one regulatory variable that does not remain simply scale-invariant when the size of the patterning field changes. Related theoretical concepts had been discussed previously under the term “modulators” and in external modulator models (Umulis and Othmer, 2013; Rasolonjanahary and Vasiev, 2016). A similar logic is also present in feedback-based models: in the expansion-repression model, an “expander” changes the characteristic length scale of the morphogen gradient, whereas in the induction-contraction model, a size-dependent inhibitor restricts signaling range (Ben-Zvi and Barkai, 2010; Almuedo-Castillo et al., 2018). Experimental studies of Sizzled-dependent BMP-Chordin scaling in *Xenopus* further demonstrated that embryo-size-dependent accumulation of a regulatory component can control morphogen degradation and thereby adjust gradient range (Inomata et al., 2013). Against this conceptual background, the Scalers Hypothesis focuses on one experimentally accessible subset of size-dependent regulatory variables: molecular regulators whose expression or activity changes with system size and thereby modulates morphogen-gradient dynamics.

## The scalers hypothesis as a unifying framework for morphogen gradient scaling

### Scaler genes as molecular size sensors

In a correctly scaled morphogenetic field, the pattern changes in size, but the relative positions of its boundaries are preserved. This means that a cell located at the same relative position in a small or large embryo should receive the appropriate level of patterning signal for that position. At first glance, one might therefore expect that the concentrations of most molecular regulators should remain similar when embryo size changes. However, theoretical and computational studies of scaling reaction-diffusion systems indicate that this is not sufficient. Accurate proportional scaling usually requires at least one regulatory component whose concentration, activity, or effective influence changes with system size. Such components were described in earlier theoretical work as modulators, reflecting their ability to adjust morphogen-gradient range or shape in response to system size (Umulis and Othmer, 2013; Rasolonjanahary and Vasiev, 2016).

This point becomes clearer when two influential feedback-based scaling models are considered from the perspective of size-dependent regulators. The expansion-repression model of BMP-Chordin scaling was proposed by Ben-Zvi and Barkai (2010), and the induction-contraction model of Nodal-Lefty scaling was proposed by Almuedo-Castillo et al. (2018). These studies showed how feedback within morphogen networks can adjust signaling range to system size. A later analysis asked a different question: are there any components in these models whose levels change when system size is reduced? This analysis indicated that Sizzled decreases in the BMP-Chordin expansion-repression model, whereas Lefty increases in the Nodal-Lefty induction-contraction model (Nesterenko and Zaraisky, 2019). Thus, when viewed from the perspective of size-dependent regulators, both previously established models contain at least one component whose level changes systematically with system size.

Large-scale computational screening of approximately 400,000 expansion-repression and induction-contraction models further demonstrated that the presence of at least one size-dependent component is a general requirement for accurate scaling within these classes of reaction-diffusion systems (Orlov et al., 2022). Moreover, formal mathematical analysis of closed reaction-diffusion systems with zero-flux boundary conditions showed that proportional scaling of morphogen gradients requires at least one component whose concentration varies with system size (Timoshina et al., 2024; see Supplementary Text S2 for the proof of the scaling lemma). In such closed autonomous systems, perfect geometric scaling is incompatible with uniform scaling of all dynamic variables.

Importantly, this constraint applies specifically to closed systems. If the pattern-forming network is open and its scaling is influenced by regulatory inputs external to the core reaction–diffusion circuit, the same logical requirement persists in a different form: at least one regulatory element must exhibit size-dependent variation. In closed systems this component must be internal to the network; in open systems it may operate from outside the core morphogenetic module. Thus, irrespective of whether scaling is implemented through intrinsic feedback architecture or external regulation, accurate proportional scaling requires the presence of at least one molecular component whose concentration changes systematically with system size. By modulating morphogen gradient dynamics in response to changes in global geometry, these components function as effective molecular size sensors.

This reasoning led to the formulation of the Scalers Hypothesis, according to which certain genes exhibit size-dependent expression and thereby regulate morphogen gradient scaling (Nesterenko and Zaraisky, 2019; Orlov et al., 2022; Timoshina et al., 2024). The identification of such genes requires experimental strategies capable of detecting robust size-dependent changes in gene expression.

It is important to emphasize that scalers do not constitute a structurally or evolutionarily defined gene family. They are not grouped on the basis of shared protein domains, biochemical activity, or molecular function. Rather, scalers represent a functional category defined at the systems level. In principle, genes encoding proteins of diverse structural classes and molecular activities may act as scalers, provided that their products regulate morphogen gradient dynamics in a size-dependent manner. What unites them is not molecular similarity but their shared role in ensuring proportional patterning.

This conceptual clarification has implications that extend beyond the specific systems analyzed to date. If correct scaling generically requires at least one size-dependent regulatory component, embryonic patterning networks may be organized hierarchically, with gradient-generating modules operating under the control of upstream size-sensitive regulators. Such hierarchical organization would represent a general design principle of morphogenetic fields, ensuring that global geometric parameters can be translated into local biochemical dynamics without destabilizing core pattern-forming interactions.

Beyond its theoretical implications, the Scalers Hypothesis also suggests a practical experimental strategy. One way to study scaling is to look for genes or proteins whose levels change when embryo size changes. These size-responsive factors can then be tested functionally to determine whether they help adjust morphogen gradients to the dimensions of the embryo or tissue. This approach does not require complete prior knowledge of the patterning network. By comparing normally sized and experimentally reduced embryos, or similarly reduced organ primordia such as the neural tube, eye primordium, or limb bud, candidate scalers can be identified from transcriptomic or proteomic differences. Their later functional analysis may reveal how size information is connected to the signaling networks that organize the field. In this way, the search for scaler genes provides a practical route for identifying molecular regulators of size-dependent patterning.

At the same time, it should be noted that the experimental strategy derived from the Scaler Hypothesis has an important limitation. It is designed to identify molecular regulators whose transcript or protein levels change with embryo or tissue size. Therefore, it would not detect purely mechanical or geometric mechanisms of scaling if these act without detectable changes in gene expression or protein abundance. For example, size-dependent changes in tissue tension, curvature, extracellular matrix organization, cell packing, fluid flow, diffusion geometry, or receptor accessibility could influence morphogen dynamics directly. Such mechanisms may function as physical regulators of scaling, but they would require different experimental approaches, including live imaging, mechanical perturbation, quantitative biophysics, and mathematical modeling.

### Experimental evidence for scalers

The Scaler Hypothesis predicts that genes whose expression changes substantially with embryo size should play functional roles in the proportional scaling of morphogen gradients. To test this prediction, a targeted screening strategy was developed based on comparative transcriptomic analysis of normally sized embryos and embryos experimentally reduced in size by separation of the first two blastomeres (Orlov et al., 2022; Timoshina et al., 2024).

### *Xenopus laevis*: mmp3 as a scaling regulator

Application of this approach in *Xenopus laevis* identified a small group of genes whose transcript levels changed significantly in half-sized mid gastrula stage embryos. Among these, the previously characterized internal scaler sizzled was detected as expected. Notably, however, the gene encoding the secreted matrix metalloproteinase Mmp3 exhibited the most pronounced size-dependent change, with expression decreasing more than tenfold in reduced embryos (Orlov et al., 2022).

Functional perturbation experiments, including morpholino-mediated knockdown and CRISPR-Cas9-mediated disruption of *mmp3*, resulted in embryos with reduced somitic mesoderm and neural plate domains. A similar reduction of these domains was observed in embryos experimentally generated by separation of the first two blastomeres. These findings indicated that Mmp3 is required for correct scaling of the BMP–Chordin system.

**FIGURE 3:**
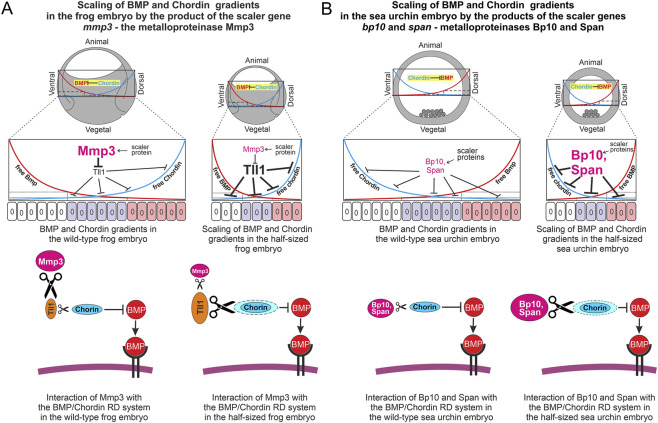
Schematic diagram of BMP-Chordin gradient scaling by scaler gene products **(A)** Scaling of BMP and Chordin gradients in the frog embryo at the early gastrula stage by the product of the scaler gene *mmp3* (Orlov et al., 2022) **(B)** Scaling of BMP and Chordin gradients in the sea urchin embryo at the mesenchymal blastula stage by the products of the scaler genes *bp10* and *span* (Timoshina et al., 2024).

Mechanistically, Mmp3 cleaves Tolloid, a metalloprotease responsible for Chordin degradation. By limiting Tolloid activity, Mmp3 indirectly stabilizes Chordin and promotes its long-range diffusion. In reduced embryos, strong downregulation of *mmp3* increases Tolloid activity, restricts Chordin diffusion, and compresses the Chordin gradient. This compression allows the opposing BMP activity gradient to rescale appropriately to the reduced embryonic dimensions. A quantitative RD model incorporating these interactions reproduced the experimentally observed scaling behavior *in silico* (Orlov et al., 2022). The molecular logic of this mechanism is summarized in Figure 3A.

Importantly, *mmp3* expression is spatially uniform and does not respond directly to BMP signaling, indicating that its regulation is independent of the BMP–Chordin RD network.

### Sea urchin: Span and bp10 as scaling regulators

A comparable screening strategy applied to the green sea urchin *Strongylocentrotus droebachiensis* identified two astacin-family metalloproteases, *span* and *blastula protease 10* (*bp10*), as candidate size-dependent regulators (Timoshina et al., 2024). During establishment of the dorsal–ventral BMP–Chordin system at the mesenchymal blastula stage, both genes were expressed approximately fourfold more strongly in reduced embryos.

In contrast to Mmp3 in *Xenopus*, Span and Bp10 directly proteolyze Chordin rather than its inhibitor Tolloid. Elevated expression of these proteases in reduced embryos promotes Chordin degradation and leads to compression of its gradient, thereby enabling appropriate rescaling of BMP signaling. Knockdown of *span* and *bp10* disrupted this adaptive adjustment, confirming their functional role in scaling (Figure 3B).

In half-sized embryos, size-dependent changes in the concentrations of secreted metalloproteinases enhance Chordin degradation by different mechanisms: indirectly through Mmp3-mediated regulation of Tolloid activity in *Xenopus*, and directly through Chordin cleavage by Bp10 and Span in sea urchins. This enhanced degradation prevents excessive Chordin accumulation that would otherwise result from the reduced size of the embryo and allows the BMP-Chordin gradients to rescale appropriately to embryo dimensions.

Although the regulatory logic differs between systems, Mmp3 levels decrease in reduced frog embryos to enhance Chordin degradation indirectly, whereas Span and Bp10 levels increase in reduced sea urchin embryos to promote Chordin degradation directly. In both cases, scaling is achieved through size-dependent modulation of Chordin turnover, leading to gradient compression in smaller embryos. Notably, similar to *mmp3*, *span* and *bp10* are expressed uniformly throughout the sea urchin embryo (Timoshina et al., 2024). It should be noted that the anatomical orientation of the BMP-Chordin system differs between amphibian and sea urchin embryos. In *Xenopus*, Chordin is produced dorsally and BMP activity is highest ventrally, whereas in sea urchin embryos Chordin is expressed on the ventral side and BMP signaling is highest dorsally. Thus, the two systems are compared here at the level of regulatory logic rather than anatomical orientation.

Together, these findings provide direct experimental validation of the theoretical prediction that accurate proportional scaling requires size-dependent regulatory components. Moreover, they demonstrate that such components can be identified through systematic comparative transcriptomic analysis and functionally confirmed *in vivo*. These results establish scaler genes not merely as theoretical constructs, but as molecular entities that integrate geometric size with morphogenetic signaling dynamics.

### Conceptual classification of scalers

**FIGURE 4:**
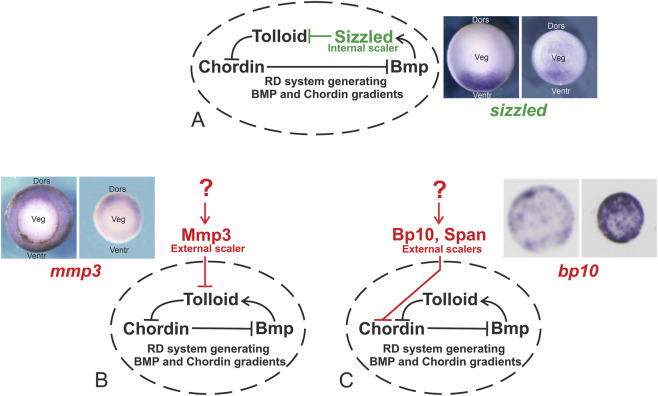
Internal and external scaling schemes **(A)** Internal scaler. *Sizzled* is an intrinsic component of the BMP–Chordin reaction–diffusion (RD) system and its expression depends on BMP signaling levels. In *Xenopus laevis* mid-gastrula embryos (wild-type and half-sized), *sizzled* is expressed asymmetrically along the dorsal–ventral axis within the blastopore marginal zone, with maximal expression on the ventral side where BMP activity is highest (Inomata et al., 2013; Orlov et al., 2022). Both embryos are shown from the vegetal side, with the ventral region oriented at the bottom **(B,C)** External scalers. **(B)** *Mmp3* is not regulated by BMP signaling and does not form part of the BMP–Chordin RD network. In *Xenopus laevis* mid-gastrula embryos (wild-type and half-sized), *mmp3* is expressed approximately uniformly around the blastopore marginal zone (Orlov et al., 2022). Embryos are shown from the vegetal side, with the ventral region oriented at the bottom **(C)** In the sea urchin *Strongylocentrotus droebachiensis*, the scaler genes *bp10* and its homolog *span* are expressed uniformly in mesenchyme blastula-stage embryos, both wild-type and reduced in size (Timoshina et al., 2024). The spatially uniform expression pattern of these genes, together with their independence from BMP–Chordin feedback regulation, supports their classification as external scalers. Scale bars: **(A,B)** 200 μm; **(C)** 25 μm.

The accumulated experimental and theoretical evidence supports a conceptual classification of scaler genes into two mechanistically distinct categories (Figure 4).

Internal scalers are embedded within reaction–diffusion networks and derive their size dependence from intrinsic feedback architecture. Their expression is directly regulated by the morphogen gradients they help shape, and they typically exhibit spatially patterned expression domains that reflect this regulatory integration. Representative examples include *sizzled* in the BMP–Chordin system and *lefty* in the Nodal–Lefty network. As illustrated in Figure 4A, Sizzled inhibits Tolloid, a protease responsible for Chordin degradation, while its own transcription is activated by BMP signaling, forming a self-regulatory feedback loop within the BMP–Chordin network. Not every component of a feedback loop should therefore be classified as an internal scaler. In this review, the term is reserved for feedback components whose concentration, activity, or effective influence changes with system size and whose perturbation contributes to proportional adjustment of morphogen-gradient dynamics.

In contrast, *mmp3*, *bp10*, and *span* differ fundamentally from previously characterized internal scalers (Figures 4B,C). Although their protein products influence Chordin turnover, either through degradation of Tolloid in the case of Mmp3 or through direct proteolysis of Chordin in the case of Bp10 and Span, they operate independently of the core BMP–Chordin reaction–diffusion circuitry.

In *Xenopus laevis*, two independent criteria support the classification of *mmp3* as an external scaler. First, experimental elevation of BMP signaling does not alter *mmp3* expression, demonstrating that it is not transcriptionally regulated by BMP activity and therefore is not embedded within the BMP–Chordin reaction–diffusion network (Orlov et al., 2022). Second, *mmp3* is expressed uniformly along the dorsal–ventral axis, in contrast to the spatially patterned expression typical of internal scalers whose transcription reflects morphogen gradient structure. Together, these observations indicate that Mmp3 modulates morphogen gradients from outside the feedback architecture that generates them.

In the sea urchin *Strongylocentrotus droebachiensis*, the classification of *span* and *bp10* as external scalers is currently supported primarily by their spatially uniform expression in both wild-type and reduced embryos (Timoshina et al., 2024). Although direct tests of BMP-dependent transcriptional regulation have not yet been performed, the absence of spatial patterning characteristic of morphogen-responsive genes strongly suggests that these proteases function outside the core BMP–Chordin regulatory network.

A notable shared property of the experimentally identified external scalers is that Mmp3, Span, and Bp10 are secreted proteases expressed broadly across the morphogenetic field in which BMP-Chordin gradients are established. From a reaction-diffusion perspective, this combination of uniform expression and proteolytic activity is well suited for gradient scaling. Because the shape and range of a morphogen gradient depend on the balance between production, diffusion, transport, and degradation, spatially homogeneous regulation of morphogen turnover provides a direct way to adjust gradient length scale without strongly distorting the overall profile (Gierer and Meinhardt, 1972; Ben-Zvi and Barkai, 2010; Umulis and Othmer, 2013). In this way, uniformly expressed secreted proteases can coordinate Chordin turnover across the whole field, either by directly cleaving Chordin, as in the case of Span and Bp10, or by acting through an intermediate regulator, as in the case of Mmp3, which cleaves the Chordin-inactivating protease Tolloid.

This mechanistic convergence may help explain why the external scalers identified so far share broad expression and proteolytic function. It also suggests that spatially uniform expression across a morphogenetic field may be a useful criterion for identifying candidate external scalers in other systems. At the same time, proteolytic activity should not be regarded as a defining feature of external scalers. In principle, molecules of different structural classes and biochemical activities could perform similar size-dependent regulatory functions if they modify morphogen production, degradation, transport, diffusion range, receptor access, or downstream interpretation in a size-dependent manner.

The existence of external scalers suggests a partial modular separation between size sensing and pattern generation: size-dependent information may be detected outside the core morphogenetic network and then transmitted to it through regulators that modify morphogen turnover, transport, or distribution. Such modular organization has important evolutionary implications. If size sensing and pattern generation are partly separable, changes in egg size, yolk content, maternal provisioning, cleavage geometry, or overall embryo dimensions could be accommodated without major restructuring of the core morphogenetic network. In this scenario, scaler genes may function as interfaces that translate global geometric parameters into controlled adjustments of morphogen dynamics. This could allow conserved pattern-forming circuits to remain functional across natural variation in embryo size and during evolutionary changes in reproductive strategy.

However, this conceptual framework raises a fundamental mechanistic question: how is embryo size detected at the molecular level, and how is geometric information converted into size-dependent regulation of scaler gene expression? While internal scalers can be explained by the intrinsic feedback logic of reaction-diffusion systems, the regulation of external scalers implies the existence of additional size-sensing mechanisms operating independently of the morphogen gradients they regulate. Identifying these mechanisms is therefore central to understanding how developing systems couple global geometry to local molecular dynamics. The following section examines possible molecular and biophysical mechanisms that could underlie such size-dependent regulation.

### Potential mechanisms of size-dependent regulation of external scalers

Because external scalers are not known to be controlled by the morphogen gradients they modulate, their size-dependent expression must depend on other features of the embryo. Genes such as *mmp3*, *span*, and *bp10* may therefore respond, directly or indirectly, to parameters such as embryo geometry, volume, surface area, mechanical state, or metabolic condition. In addition, reduced embryos may differ in total cell number, cell size, tissue packing, or nuclear-to-cytoplasmic ratio. These parameters could influence morphogen production, diffusion geometry, mechanical state, or transcriptional responses, and therefore may contribute to size-dependent regulation. At present, no definitive molecular mechanism for such size detection has been established. The possibilities discussed below should therefore be regarded as conceptual scenarios rather than experimentally validated pathways. Nevertheless, outlining plausible mechanisms is useful because it generates testable predictions for future work.

**FIGURE 5:**
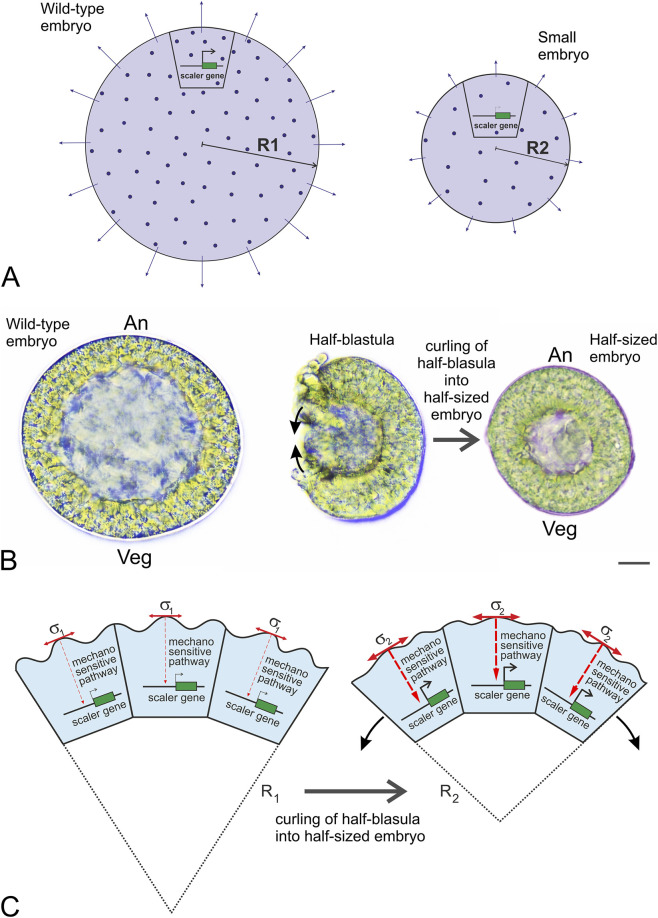
Conceptual models for size-dependent regulation of external scalers **(A)** Hypothetical biochemical size-sensing mechanism. A diffusible molecule X (black dots) is produced uniformly throughout the embryonic volume and removed across the surface. Larger embryos (radius R1) contain greater volume relative to surface area than smaller embryos (radius R2). Under steady-state conditions, the average concentration of X is expected to scale with the ratio of volume to surface area and therefore with embryo linear dimension. If transcription of a scaler gene depends on the concentration of such a molecule, size-dependent expression could arise without direct measurement of geometric parameters. This scheme represents a conceptual illustration rather than an experimentally validated mechanism **(B)** When developing from one of the separated first two blastomeres, the sea urchin embryo first forms a cup-shaped half-blastula, which then curls into a whole half-size blastula. Scale bar 20 μm. **(C)** Hypothetical mechanical size-sensing mechanism. During curling of the sea urchin half-blastula into a smaller spherical blastula, mechanical stress in the ectodermal layer increases (σ2 > σ1) as curvature becomes higher (R2 < R1), consistent with general mechanical principles. Such differences in stress may modulate mechanosensitive signaling pathways, which in turn regulate scaler gene expression. This scheme illustrates how mechanical cues could couple global geometry to transcriptional control independently of morphogen gradients.

One conceptually simple possibility involves diffusible biochemical factors whose steady-state concentration depends on embryo geometry. In this hypothetical scheme, a small molecule X is produced throughout the embryonic volume and removed or exchanged across the embryonic surface. Under steady-state conditions, the average concentration of X could scale with the ratio of embryo volume to surface area and therefore with embryo linear dimensions (Figure 5A). Larger embryos would be expected to maintain higher equilibrium concentrations of X than smaller embryos. If transcription of an external scaler depended on the concentration of such a factor, size-dependent gene expression could arise without direct measurement of embryo dimensions.

Although abstract, this framework illustrates how biochemical size sensing might operate at the whole-embryo level. At present, no specific molecule has been shown to behave in this way during embryonic scaling. Candidate mediators would need to satisfy two main conditions: their production or accumulation should depend on embryonic volume, and their removal, exchange, or dilution should depend on surface area or geometry. Identifying such molecules remains an open experimental problem. Intracellular pathways responsive to metabolic, redox, or signaling states could, in principle, couple such biochemical cues to transcriptional regulation of scaler genes, but direct evidence linking specific biochemical factors to size-dependent regulation of external scalers is currently lacking.

An alternative, and not mutually exclusive, possibility involves mechanical size sensing. Embryos of different sizes differ not only in volume but also in curvature, tissue tension, and mechanical stress distribution. For example, a sea urchin embryo developing from one of the first two separated blastomeres initially forms a cup-shaped half-blastula, which subsequently curls into a smaller spherical embryo (Figure 5B) (Driesch, 1891; Orlov et al., 2025; Suzuki et al., 2025). Such geometric transformations are expected to alter mechanical stress within the ectodermal layer as curvature increases. In smaller embryos, higher curvature may generate elevated mechanical strain within epithelial sheets, consistent with basic principles of tissue mechanics. Such curvature-dependent differences in stress could, in principle, activate mechanosensitive signaling pathways and thereby influence gene expression (Figure 5C).

Recent advances in mechanobiology have identified multiple molecular systems capable of converting mechanical cues into transcriptional responses. These include mechanosensitive ion channels (Coste et al., 2010; Li et al., 2014), RhoA–ROCK signaling (Acharya et al., 2018; Boyle et al., 2020), focal adhesion kinase (Lachowski et al., 2018; Zhou et al., 2022), and MAP kinase pathways such as ERK1/2 (Pereira et al., 2011; Bailey et al., 2019; De Belly et al., 2021; Munne et al., 2021). Among transcriptional regulators, YAP, a key effector of the Hippo pathway, functions as a prominent mechanosensitive integrator. YAP nuclear localization correlates with cytoskeletal tension, substrate stiffness, and cell density (Dupont et al., 2011; Wada et al., 2011; Dasgupta and McСollum, 2019; Ahmad et al., 2022), and mechanical cues influence developmental processes such as lineage segregation in the mammalian blastocyst (Maître et al., 2016) and neural crest competence in *Xenopus laevis* (Alasaadi et al., 2024).

Similarly, mechanical perturbations in *Xenopus* embryos can activate ERK1/2 signaling independently of canonical ligand stimulation, leading to cytoskeletal remodeling and altered tissue stiffness (Kinoshita et al., 2020). These observations demonstrate that embryonic tissues are capable of translating mechanical inputs into gene regulatory responses.

Importantly, mechanical size sensing need not operate only through transcriptional regulation of scaler genes. Size-dependent changes in curvature, tissue tension, extracellular matrix organization, cell packing, or tissue stiffness could also influence morphogen dynamics directly, for example, by altering extracellular diffusion, ligand transport, receptor accessibility, endocytosis, tissue flows, or morphogen clearance (Lander, 2007; Kicheva et al., 2012; Hannezo and Heisenberg, 2019; Goodwin et al., 2021; Caldarelli et al., 2024). Such mechanisms would not necessarily produce detectable changes in scaler gene expression, but could nevertheless contribute to proportional patterning.

Whether biochemical or mechanical mechanisms regulate expression of external scalers during embryonic scaling remains to be determined. It is also possible that multiple inputs act in combination, integrating geometric, metabolic, and mechanical information. Dissecting how global size parameters are sensed and translated into transcriptional control of scaler genes, or directly into changes in morphogen dynamics, represents a critical next step for testing and refining the Scalers Hypothesis.

## Discussion

The examples discussed in this review indicate that embryonic scaling is not produced by a single universal mechanism. Proportional patterning can arise through several routes, including feedback-controlled morphogen-gradient regulation, size-dependent molecular inputs, maternal control of morphogen production, mechanical self-organization, growth, and tissue remodeling (Ben-Zvi and Barkai, 2010; Inomata et al., 2013; Cheung et al., 2011; Almuedo-Castillo et al., 2018; Caldarelli et al., 2024; Hill and Petersen, 2015). The value of the scaler-gene framework is that it focuses on one experimentally accessible part of this broader problem: molecular regulators whose expression, concentration, or activity changes with embryo or tissue size and thereby helps adjust patterning mechanisms to the dimensions of the system.

This framework does not replace classical morphogen-gradient models. Rather, it asks how morphogen systems acquire information about the size of the field in which they operate. Related theoretical concepts had been discussed previously under the term “modulators” and in external modulator models (Umulis and Othmer, 2013; Rasolonjanahary and Vasiev, 2016). The Scalers Hypothesis addresses this problem from an experimentally oriented perspective by focusing on concrete genes and proteins whose size-dependent expression or activity can be identified, perturbed, and functionally tested in embryos. In this sense, scaler genes provide a practical route from theoretical models of scaling to experimentally tractable molecular mechanisms.

A formal mathematical result further strengthens this view. For closed reaction-diffusion systems with zero-flux boundary conditions, ideal proportional scaling is impossible if all internal dynamic components remain scale-invariant. At least one internal component must change with system size (Timoshina et al., 2024). Real embryonic systems are not strictly closed, and therefore size-dependent regulation may also be supplied by external inputs acting on the core gradient-forming network. In such open systems, internal scalers may be complemented by external scalers, or in some cases functionally replaced by them. Thus, accurate scaling requires at least one size-dependent regulatory variable, but this variable may be embedded within the morphogenetic network, act from outside it, or involve both levels of regulation. This theoretical constraint explains why identifying scalers and their mechanisms of action is central to understanding embryonic scaling.

The distinction between internal and external scalers is useful because it separates two different ways in which size dependence can enter a patterning system. Internal scalers, such as Sizzled or Lefty, derive their size dependence from feedback within the morphogenetic network itself. External scalers, such as Mmp3 in *Xenopus* and Span/Bp10 in sea urchins, appear to be regulated outside the core BMP-Chordin network and modulate that network from the outside (Inomata et al., 2013; Almuedo-Castillo et al., 2018; Orlov et al., 2022; Timoshina et al., 2024). This distinction does not imply that all scaling mechanisms must fall neatly into two categories. Rather, it provides a working classification that helps define experimentally testable questions: which components change with size, whether they are controlled by the morphogen network itself, and how their perturbation alters gradient range and pattern proportions.

A modular separation between size sensing and pattern generation may have important evolutionary consequences. Embryos naturally vary in size because of differences in egg size, yolk content, maternal provisioning, cleavage geometry, and reproductive strategy. In *Xenopus laevis*, for example, embryos from a single clutch may vary substantially in diameter while still preserving proportional patterning (Leibovich et al., 2020). Across evolution, egg size and early embryo geometry can change, whereas core patterning pathways such as BMP, Nodal, Wnt, and FGF are deeply conserved. If size-sensitive regulators can adjust the parameters of these conserved networks, then embryo size may evolve without requiring complete rewiring of the underlying pattern-forming circuitry. External scalers could therefore act as interfaces between global physical properties of the embryo and conserved local signaling systems. This modularity may increase developmental robustness and evolvability by allowing size-related traits and patterning mechanisms to vary semi-independently.

The same logic may apply beyond early embryonic axes. In organ primordia, local morphogen gradients often pattern fields that vary in size during growth or between individuals. Neural tubes, limb buds, eyes, and other primordia must coordinate patterning with tissue expansion, shape changes, and mechanical constraints. It remains unknown whether these systems use molecular regulators analogous to embryonic scalers, but they provide promising contexts in which the scaler framework could be tested. A practical approach would be to compare normally sized and experimentally reduced primordia, identify size-responsive genes or proteins, and then test whether these candidates regulate morphogen range, degradation, transport, receptor accessibility, or downstream threshold responses (Lander, 2007; Rogers and Schier, 2011; Kicheva et al., 2012).

Regenerative systems provide another important context for size-dependent patterning. In *Hydra*, small tissue fragments can regenerate complete axes with appropriate proportions, and in planarians, fragments of very different sizes can restore axial polarity, organ proportions, and body pattern through a combination of positional information, growth, and remodeling (Bode and Bode, 1980; Hill and Petersen, 2015). These systems are not expected to use exactly the same mechanisms as early embryos. Nevertheless, they face a similar problem: a patterning system must operate in a field whose size and geometry have changed. The scaler framework may therefore be useful if applied broadly, not as a claim that the same genes act in regeneration, but as a way to ask whether particular molecular or physical variables couple fragment size to organizer activity, morphogen range, growth rate, or tissue remodeling.

Engineered multicellular systems offer a complementary experimental opportunity. Gastruloids and related embryoid models can be generated with controlled initial cell numbers, aggregate sizes, and mechanical environments. This makes them especially useful for testing whether size-dependent regulators exist and how they interact with self-organization. For example, changing initial aggregate size while measuring morphogen activity, mechanical state, and transcriptomic responses could help distinguish between molecular scalers, mechanical scalers, and purely geometric constraints. Such systems may therefore provide a bridge between embryo-based studies and synthetic approaches to developmental patterning (Fiuza et al., 2025).

Several limitations of the current scaler-gene approach should be emphasized. First, transcriptomic and proteomic screens can identify candidates whose RNA or protein levels change with size, but they will not detect purely mechanical mechanisms, changes in transport geometry, receptor accessibility, protein activity, or post-translational regulation if these occur without changes in abundance. Second, size-dependent expression alone does not demonstrate scaler function. Candidate scalers require functional perturbation, rescue experiments, and direct analysis of morphogen-gradient dynamics. Third, experimentally reduced embryos may not fully reproduce all aspects of natural size variation, especially when natural differences arise during oogenesis or maternal provisioning. These limitations mean that scaler screens should be viewed as entry points into mechanism, not as complete explanations of scaling.

Mechanical mechanisms deserve particular attention in this context. Size-dependent changes in curvature, tissue tension, extracellular matrix organization, cell packing, fluid flow, or tissue stiffness could influence patterning directly, without first changing scaler gene expression. Such effects may alter extracellular diffusion, ligand transport, receptor accessibility, endocytosis, morphogen clearance, or tissue movements. More generally, developmental systems often involve feedback between mechanical and biochemical processes, and recent work in avian embryos shows that tissue mechanics can itself contribute to embryonic regulation (Hannezo and Heisenberg, 2019; Goodwin et al., 2021; Caldarelli et al., 2024). Thus, scaler genes should be considered one component of a broader size-sensing landscape that may also include mechanical and geometric mechanisms.

Future progress will require combining several approaches. Comparative transcriptomics and proteomics can identify candidate molecular regulators, but these data should be integrated with live imaging of morphogen gradients, quantitative analysis of tissue mechanics, perturbation of candidate genes, and mathematical modeling. Comparative studies across species with different egg sizes or developmental geometries may reveal whether scaler-like mechanisms are conserved or repeatedly evolved. Similarly, applying controlled size perturbations to organ primordia, regenerating tissues, and gastruloids could test whether modular size sensing is a general principle of multicellular patterning.

In summary, embryonic scaling provides a powerful system for studying how global geometry is translated into local developmental decisions. The Scalers Hypothesis contributes to this field by defining a class of molecular regulators that connect embryo size to morphogen-gradient dynamics. At the same time, scaling is broader than scaler genes alone. It likely emerges from the integration of biochemical gradients, mechanical forces, growth, metabolism, and tissue geometry. Understanding how these layers interact will be essential for explaining how embryos and regenerating systems preserve proportional form despite variation in size.
